# Sero-identification of the aetiologies of human malaria exposure (*Plasmodium* spp.) in the Limu Kossa District of Jimma Zone, South western Ethiopia

**DOI:** 10.1186/s12936-019-2927-3

**Published:** 2019-08-27

**Authors:** Sindew Mekasha Feleke, Bokretsion Gidey Brhane, Hassen Mamo, Ashenafi Assefa, Adugna Woyessa, Guilherme Maerschner Ogawa, Vitaliano Cama

**Affiliations:** 1 Ethiopian Public Health Institute (EPHI), Addis Ababa, Ethiopia; 2 Department of Microbial, Cellular and Molecular Biology, College of Natural Sciences, Addis Ababa University, Addis Ababa, Ethiopia; 3 Division of Parasitic Diseases and Malaria, Centers for Disease Control and Prevention (CDC), Atlanta, GA USA

**Keywords:** Malaria, Sero-diagnosis, IgG antibodies, *Plasmodium* species, Multiplex bead assay, Ethiopia

## Abstract

**Background:**

Malaria remains a very important public health problem in Ethiopia. Currently, only *Plasmodium falciparum* and *Plasmodium vivax* are considered in the malaria diagnostic and treatment policies. However, the existence and prevalence of *Plasmodium ovale* spp. and *Plasmodium malariae* in Ethiopia have not been extensively investigated. The objective of this study was to use a multiplex IgG antibody detection assay to evaluate evidence for exposure to any of these four human malaria parasites among asymptomatic individuals.

**Methods:**

Dried blood spots (DBS) were collected from 180 healthy study participants during a 2016 onchocerciasis survey in the Jimma Zone, southwest Ethiopia. IgG antibody reactivity was detected using a multiplex bead assay for seven *Plasmodium* antigens: *P. falciparum* circumsporozoite protein (CSP), *P. falciparum* apical membrane antigen-1 (AMA1), *P. falciparum* liver stage antigen-1 (LSA1), and homologs of the merozoite surface protein-1 (MSP1)-19kD antigens that are specific for *P. falciparum*, *P. vivax*, *P. ovale* spp. and *P. malariae.*

**Results:**

One hundred six participants (59%) were IgG seropositive for at least one of the *Plasmodium* antigens tested. The most frequent responses were against *P. falciparum* AMA1 (59, 33%) and *P. vivax* (55, 28%). However, IgG antibodies against *P. ovale* spp. and *P. malariae* were detected in 19 (11%) and 13 (7%) of the participants, respectively, providing serological evidence that *P. malariae* and *P. ovale* spp., which are rarely reported, may also be endemic in Jimma.

**Conclusion:**

The findings highlight the informative value of multiplex serology and the need to confirm whether *P. malariae* and *P. ovale* spp. are aetiologies of malaria in Ethiopia, which is critical for proper diagnosis and treatment.

## Background

In 2017, an estimated 219 million cases of malaria occurred worldwide, compared with 239 million cases in 2010 and 217 million cases in 2016 [1]. An estimated 435,000 deaths from malaria recorded globally in 2017, compared with 451,000 estimated deaths in 2016, and 607,000 in 2010 [1]. An increasing number of countries are moving towards eliminating malaria. Ethiopia is among the sub-Saharan African countries that have successfully reduced their malaria burden in the last decade [2, 3]. The Government of Ethiopia launched an elimination strategy taking advantage of this progress, in line with the commitment of African leaders to attain malaria elimination by 2030 [4]. The Federal Ministry of Health (FMoH) achieved significant declines in malaria mortality and incidence and has recently declared its objective to eliminate malaria by 2020 in low transmission areas that have an annual parasite incidence (API) of less than one per 1000 total populations. The goal is to achieve nationwide elimination by 2030 [4]. The *Plasmodium* species causing malaria in Ethiopia are *Plasmodium falciparum*, accounting for about 60% of cases, and *Plasmodium vivax*, which accounts for about 40% of cases, with the former being the cause of most severe clinical manifestations and deaths [4, 6]. Current case management protocols target both species [4, 5], but may not be adequate for other *Plasmodium* species.

*Plasmodium ovale* spp. [7, 8] and *Plasmodium malariae* [6] have also been reported from limited areas in Ethiopia. Additionally, the FMoH has historical records that include reports of sizeable number of cases of both *P. malariae* and *P. ovale* spp. during the 1980s and 1990s [6–8]. However, current information about population-level exposure estimates to these species is limited.

The multiplex bead assay (MBA) can provide accurate and quantitative information about the diversity of exposure to malaria parasites by measuring human antibody levels against several antigens concurrently. In this study, MBA incorporating seven *Plasmodium* antigen targets used to assess malaria seroprevalence in the Limu Kossa District, Jimma Zone in southwest Ethiopia. Four antigen targets were specific for *P. falciparum* infection, including indicators of long and short-term infection. Homologs of the *P. falciparum* merozoite surface protein 1 *(*MSP-1) for *P. vivax*, *P. ovale* spp. and *P. malariae* were used to characterize species-specific responses [9–13].

## Methods

### Study area and design

A total of 180 dried blood spot (DBS) samples were collected in October 2016 as part of a community-based onchocerciasis survey in the villages of Arengama 1, Arengama 2 and Konche in Limu Kossa District, Jimma zone, Oromia Region, which is located 400 km in the south west of Addis Ababa. Study participants were selected through convenience sampling among village residents with age 5 years and above who provided consent and assent and participated in an interview and finger prick blood collection.

### Laboratory analysis by multiplex bead assay (MBA)

Seven malaria antigens were coupled to polystyrene beads using 1-ethyl-3-(3-dimethylaminopropyl) (Calbiochem, Woburn, MA) carbodiimide to convert carboxyl groups on the surface of the magnetic microspheres (MagPlex Beads; Luminex Corporation, Austin, TX) to reactive esters. The esters readily react with available primary amino groups on the antigens to form covalent amide bonds between antigen and microspheres. The seven recombinant antigens were expressed as Glutathione S-transferases (GST) fusion proteins and were coupled to activated beads in separate reaction tubes (12.5 million beads each) using 30 µg *P. falciparum* CSP, 60 µg *P. falciparum* LSA1, 20 µg *P. falciparum* AMA-1, and 20 µg of each of the four species-specific MSP-1-19kD antigen in 50 mM 2-(N-morpholino) ethanesulfonic acid (MES), 0.85% NaCl, pH 5.0. As a control, for non-specific binding of human IgG, 15 µg GST was coupled to beads a using the same conditions.

The MBA was conducted using serum eluted from DBS to a final dilution of 1:400 in buffer B (PBS with 0.5% BSA, 0.3% Tween 20, 0.1% sodium azide, 0.5% polyvinyl alcohol, 0.8% polyvinylpyrrolidone, 0.1% casein, and 4 µg/ml crude *Escherichia coli* extract), incubated for 1 h at 37 °C, and then stored at 4 °C overnight. The next day, the assay plate (Sigma Millipore, MAHVN4550, Burlington, MA) was pre-wetted with 200 µl phosphate buffered saline pH 7.2 plus 0.05% Tween20 (PBS-T) and evacuated with vacuum. To prepare a bead mix, beads coupled with each of the 7 different malaria antigens were re-suspended by vortexing, and 15 µl of each (~ 250,000 beads) of the antigen-coupled beads were transferred to conical tubes containing 5 ml Buffer A (PBS-T, 0.5% bovine serum albumin, 0.02% NaN_3_). Of this bead mix, 50 µl were transferred to each well (~ 2500 each bead classification per well) of the assay plate using multichannel pipette and washed twice with 100 µl of PBS-T. To detect IgG reactivity, 50 µl of the prepared samples were added in duplicate wells, incubated for 90 min at room temperature on a shaker and then washed three times. After washing, 50 µl of biotinylated mouse anti-human IgG (Southern Biotech, 9042-08, Birmingham, USA) diluted at 50 ng/well in buffer A was added to each well and incubated for 45 min at room temperature on a shaker. After three washes to remove the excess biotinylated antibodies, 50 µl 1:200 streptavidin-phycoerythrin (Invitrogen S866, Waltham, USA) solution in buffer A was added per well and incubated at room temperature for 1 h on a shaker. The plate was washed once, 50 µl of buffer A were added to each well and incubated for 30 min at room temperature on a shaker. In the last step, the plate was washed and 125 µl of PBS added to each well, incubated for 2 min with shaking, and followed by immediate reading on the Bio-Rad Bio-Plex 200 System, (Bio-Rad, Hercules, CA, USA). Data were acquired with Bio-Plex manager 6.1 Software (Bio-Rad) with a protocol to read plates at High RPI and 100 beads/well per region. The means of the median fluorescence intensity (MFI) minus background from blank wells (MFI-bg) were calculated as the final MFI-bg reading for duplicate wells for each sample. Positivity to the seven antigens for the four *Plasmodium* species was evaluated using previously determined cut-off values (Table 1). The cut-off was calculated using a panel of 92 samples from United States residents without history of travel outside the US. From the results, the log normal mean was calculated and added three standards deviations to get the MFI-Bg cut off.

**Table 1:** Antigens used in this study, describing the species reactivity and the corresponding cutoff values for MBA evaluation of DBS

S. no.	Antigen name	Target species	Signal cut off values (MFI-bg)
1	MSP 1-19kD	*P. vivax*	325
2	AMA 1	*P. falciparum*	558
3	MSP 1-19kD	*P. falciparum*	183
4	CSP	*P. falciparum*	449
5	LSA1	*P. falciparum*	124
6	MSP 1-19kD	*P. malariae*	450
7	MSP 1-19kD	*P. ovale* spp.	263

### Statistical analysis

Data were imported into an Excel database and analysed using SPSS V 20. Descriptive statistics were used to describe the demographic characteristics and prevalence of antibodies to each antigen individually and in combination.

## Results

### Sero-diagnosis of *Plasmodium* species

**Fig. 1:**
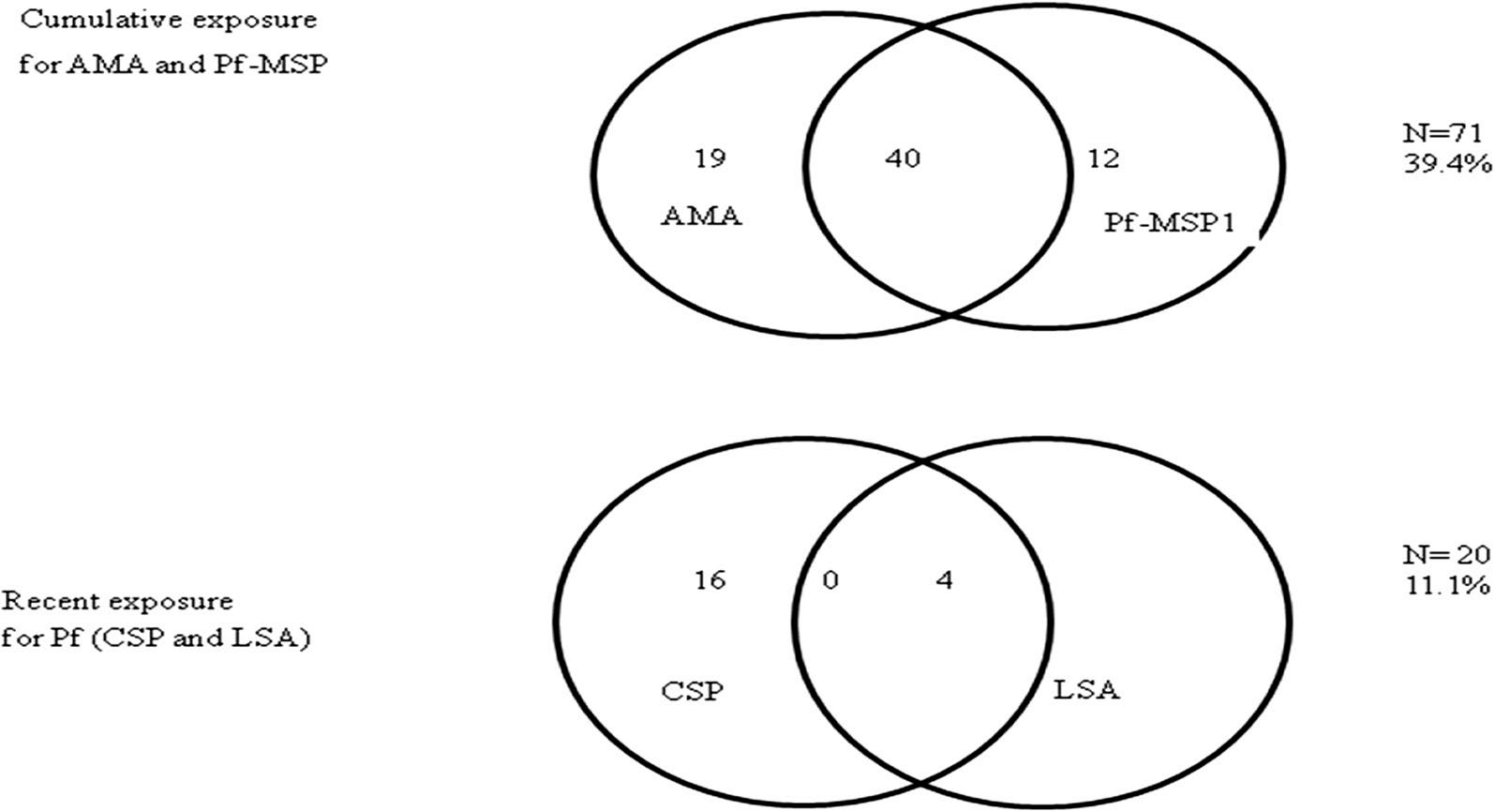
Positive cases against *Plasmodium falciparum* antigens

Of the 180 enrolled study participants, 121 (67.2%) were male and 59 (32.8%) females with a mean age of 49 years (standard deviation = 14). Of all samples analysed, 106 (59%) were seropositive for IgG antibodies against at least one of the malaria antigens tested. Antibodies reactive with *P. falciparum* were detected most frequently, with 59 (33%) seropositive persons to *P. falciparum* AMA1, 52 (29%), to *P. falciparum* MSP 1-19kD, 16 (9%) to, *P. falciparum* CSP and 4 (2%) to *P. falciparum* LSA1 (Table 2 and Fig. 1). The IgG positivity for *P. falciparum* MSP 1-19kD and/or *P. falciparum* AMA1 antigens was 39% (n = 71) and 11% (20) had evidence of exposure to either *P. falciparum* CSP and/or *P. falciparum* LSA1 antigens.

**Table 2:** Results of testing for antibodies to malaria antigens

Malaria species	Target antigen (n)	Positive (%)
*P. vivax*	MSP 1-19kD	50 (28)
*P. falciparum*	MSP 1-19kD	52 (29)
*P. malariae*	MSP 1-19kD	19 (11)
*P. ovale*	MSP 1-19kD	13 (7)
*P. falciparum*	AMA 1	59 (33)
*P. falciparum*	CSP	16 (9)
*P. falciparum*	LSA1	4 (2)

### Malaria exposure history

Fifteen percent (27 of 180) of the study participants had evidence for a lifetime exposure history to both *P. falciparum* and *P. vivax.* Additionally 6.7% had evidence for *P. falciparum* and *P. malariae* exposure. The serologic evidence of exposure to different *Plasmodium* species is indicated in Table 3.

**Table 3:** Multiplicity of malaria exposures based on IgG antibody reactivity to species specific homologs of the *Plasmodium* MSP 1-19kD antigen (n = 180)

Species for IgG positivity	Frequency	Percent (%)
*P. falciparum*+ and *P. vivax*+	27	15
*P. falciparum*+ and *P. ovale*+	6	3
*P. falciparum*+ and *P. malariae*+	12	7
*P. vivax*+ and *P. ovale*+	7	4
*P. vivax*+ and *P. malariae*+	7	4

## Discussion

Malaria elimination is the interruption of local transmission. This is achieved by detecting every infection and managing each case properly [4]. Identifying the *Plasmodium* species type and intensive implementation of malaria diagnosis and treatment accordingly is important for the elimination programmes. Malaria in Ethiopia has traditionally considered to be caused primarily by *P. falciparum* and *P. vivax* hence these species are targeted in the national malaria diagnosis and treatment strategy; there has been limited information on the importance of *P. ovale* spp. and *P. malariae* in Ethiopia. Here, a novel MBA, a bead-based technology that allows multi-analyte profiling, where microsphere beads with unique light spectra are coupled to specific antigens [12], to determine the diversity of exposure to malaria parasites.

Human infections with *Plasmodium* species elicit antibody responses to multiple parasite stages and the presence of these antibodies can be used as indicators of parasite exposure [15]. MBA using seven informative malaria antigens provided information on the *Plasmodium* species exposure types in Jimma area. The information will inform national malaria diagnosis and treatment strategies, for example, by including guidance for supporting diagnosis of all malaria species found in Ethiopia.

Antibodies to *Plasmodium* antigens such as AMA1 [14], CSP [15], MSP1 [16], and LSA-1 have all been used as biomarkers of malaria transmission in various endemic settings [11], and were included in this multiplex serology assay. The observed low seroprevalence to the non-erythrocytic antigens, CSP and LSA-1 could suggest that the study area has low transmission rates of *P. falciparum,* as antibody responses to these antigens decay faster than responses to blood-stage antigens. The MSP-1 antigen, a predominant merozoite surface antigen that is present in all examined *Plasmodium* species, and the species-specific 19kD fragments (MSP 1-19kD) were useful in this study to determine that all four species were being transmitted. *P. falciparum* and *P. vivax* MSP-1 are used extensively as serological biomarkers, and often used for population studies [17, 18] and according to a previous study, there is no evidence of cross reactivity between the species-specific MSP-1 antigens [19].

Currently, *P. falciparum* and *P. vivax* are considered the main aetiologies of malaria in Ethiopia and the national diagnostic and treatment strategies only cover these two species. However, in this survey antibody evidence in 10.6% and 7.2% of the study participants had IgG responses that were specific for *P. malariae* and *P. ovale* spp., respectively. The samples tested came from a convenience sample of the population, and thus may not be representative of the local or national population. Furthermore, because these are antibody responses, these rates may not be accurate estimates of current incidence or prevalence even in the tested population, the results clearly indicates that at some time point, *P. malariae* and *P. ovale* spp. have been present in Ethiopia. Additional studies are needed to demonstrate the distribution and prevalence for *P. malariae* and *P. ovale* spp. Even though the findings are novel for Ethiopia, both *P. ovale* spp. and *P. malariae* were similarly reported in some countries in Africa [16, 20, 21].

The study also showed the value of MBA serology, as it allows analysing multiple antibodies at one time and indicated antibody exposure to more than one human malaria species in 27% of the study participants. The seroprevalence of *P. falciparum* and *P. vivax* MSP 1-19kD antigens was almost equal, with 29 and 28% of the population in Limu Kossa having IgG antibodies to these two antigens, respectively. Fifteen percent of the population had IgG to both of these antigens, confirming that exposure to both species has occurred in Ethiopia.

While *P. falciparum* and *P. vivax* infections remain as a primary concern in Ethiopia, the detection of antibodies specific against *P. malariae* and *P. ovale* spp. is important [18] because both can cause chronic nephritic syndrome, leading to adverse reactions during treatment and high mortality rates [13]. It is challenging to diagnose *P. malariae* and *P. ovale* by microscopy due to low parasitaemia and resemblance to *P. vivax* particularly in cases of mixed infection. Furthermore, RDTs have limited ability to detect these species [22], which also leads to misdiagnosis. Inability to accurately diagnose and promptly treat *P. malariae* and *P. ovale* cases can affect patient medical outcomes in particular and the elimination programme in general. The choice of treatment for confirmed *P. ovale* spp. and *P. malariae* infection is chloroquine at 25 mg/kg for 3 days [23]. *P. ovale* spp. and *P. malariae* are also susceptible to artemisinin-based combination therapy (ACT). Mixed infection of *P. falciparum* with *P. ovale* spp. or *P. malariae* can successfully be treated with ACT drugs [23]. However, the efficacy of these drugs to *P. ovale* spp. and to *P. malariae* is not studied in Ethiopia.

Multiple serological data sets generated using MBA, which might not have otherwise been collected as part of surveillance, is helpful for program decision-making. It is important for the national treatment guidelines to explicitly include *P. malariae* and *P. ovale* spp. in the treatment algorithm; for example, microscopy might be recommended in cases where RDTs are negative but symptoms persist. Future studies with larger populations that include microscopy or DNA based methods are needed to unequivocally demonstrate the presence of active *P. malariae* and *P. ovale* infections. These findings are important for the Ethiopian programme in their quest to eliminate human malaria.

### Limitation of the study

The study participants are residents of Konche, Arengama 1 and Arengama 2 villages of Limu Kossa Districts selected based on convenient sampling. Therefore, the result may not be generalizable to the entire district population.

## Conclusion

The study showed well-defined evidence of malaria infections among the study participants, with *P. falciparum* and *P. vivax* having the highest exposure rates. It also revealed that *P. malariae* and *P. ovale* spp. were endemic at low levels in the Limu Kossa district of Ethiopia. Therefore, there is a need for robust studies to identify the distribution of *Plasmodium* species present in Ethiopia. Those findings may impact the national strategies for elimination, including clinical diagnosis and treatment, as well as the priorities for programmatic research.

## Data Availability

The database analysed in this study are available from the corresponding author.
